# Plasma Phosphorylated Tau 217 Cutoffs for Amyloid Pathology and Kidney Function, Body Mass Index, and Anemia

**DOI:** 10.1001/jamaneurol.2025.5530

**Published:** 2026-02-02

**Authors:** Jihwan Yun, Jungah Lee, Daeun Shin, Eun Hye Lee, Jun Pyo Kim, Hongki Ham, Yuna Gu, Min Young Chun, Sung Hoon Kang, Hee Jin Kim, Duk L. Na, Ko Woon Kim, Si Eun Kim, Yeshin Kim, Jaeho Kim, Na-Yeon Jung, Yeo Jin Kim, Soo Hyun Cho, Jin San Lee, Seonghyeon Kim, Henrik Zetterberg, Kaj Blennow, Fernando Gonzalez-Ortiz, Nicholas J. Ashton, Joel B. Braunstein, Philip B. Verghese, Tim West, Matthew R. Meyer, Sang Won Seo, Hyemin Jang

**Affiliations:** 1 Department of Neurology, Kyung Hee University Hospital, Kyung Hee University College of Medicine, Seoul, Republic of Korea; 2 Alzheimer’s Disease Convergence Research Center, Samsung Medical Center, Seoul, South Korea; 3 Department of Neurology, Samsung Medical Center, Sungkyunkwan University School of Medicine, Gangnam-gu, Seoul, South Korea; 4 Department of Neurology, Yonsei University College of Medicine, Seoul, South Korea; 5 Department of Neurology, Yongin Severance Hospital, Yonsei University Health System, Yongin, South Korea; 6 Department of Neurology, Korea University Guro Hospital, Korea University College of Medicine, Seoul, South Korea; 7 Department of Health Sciences and Technology, SAIHST, Sungkyunkwan University, Seoul, South Korea; 8 Department of Digital Health, SAIHST, Sungkyunkwan University, Seoul, South Korea; 9 Department of Intelligent Precision Healthcare Convergence, Sungkyunkwan University, Suwon, South Korea; 10 Happymind Clinic, Seoul, South Korea; 11 Department of Neurology, Jeonbuk National University Medical School and Hospital, Jeonju, Korea; 12 Department of Neurology, Inje University College of Medicine, Haeundae Paik Hospital, Busan, Korea; 13 Department of Neurology, Kangwon National University College of Medicine, Chuncheon-si, Gangwon-do, Korea; 14 Department of Neurology, Dongtan Sacred Heart Hospital, Hallym University College of Medicine, Hwaseong, Republic of Korea; 15 Department of Neurology, Pusan National University Yangsan Hospital, Pusan National University School of Medicine, Yangsan, Korea; 16 Department of Neurology, Kangdong Sacred Heart Hospital, Seoul, Republic of Korea; 17 Department of Neurology, Chonnam National University Medical School and Chonnam National University Hospital, Gwangju, Korea; 18 Department of Psychiatry and Neurochemistry, Institute of Neuroscience and Physiology, the Sahlgrenska Academy at the University of Gothenburg, Gothenburg, Sweden; 19 Clinical Neurochemistry Laboratory, Sahlgrenska University Hospital, Gothenburg, Sweden; 20 Department of Neurodegenerative Disease, University College London Institute of Neurology, London, United Kingdom; 21 UK Dementia Research Institute at University College London, London, United Kingdom; 22 Hong Kong Center for Neurodegenerative Diseases, Clear Water Bay, Hong Kong, China; 23 Wisconsin Alzheimer’s Disease Research Center, University of Wisconsin School of Medicine and Public Health, University of Wisconsin–Madison, Madison; 24 Paris Brain Institute, ICM, Pitié-Salpêtrière Hospital, Sorbonne University, Paris, France; 25 Neurodegenerative Disorder Research Center, Division of Life Sciences and Medicine, and Department of Neurology, Institute on Aging and Brain Disorders, University of Science and Technology of China and First Affiliated Hospital of USTC, Hefei, P.R. China; 26 Institute of Psychiatry, Psychology and Neuroscience, Maurice Wohl Clinical Neuroscience Institute, King’s College London, London, United Kingdom; 27 NIHR Biomedical Research Centre for Mental Health and Biomedical Research Unit for Dementia at South London and Maudsley NHS Foundation, London, United Kingdom; 28 Centre for Age-Related Medicine, Stavanger University Hospital, Stavanger, Norway; 29 C_2_N Diagnostics LLC, St Louis, Missouri; 30 Department of Neurology, Asan Medical Center, University of Ulsan College of Medicine, Seoul, Republic of Korea

## Abstract

**Importance:**

Plasma phosphorylated p-tau 217 levels vary with biological factors such as kidney dysfunction, body mass index (BMI), and anemia. It remains unclear whether a biological subgroup–specific optimal cutoff or a double-cutoff strategy could enhance diagnostic accuracy and cost efficiency beyond the standard single cutoff.

**Objective:**

To compare the diagnostic and economic performance of 3 plasma p-tau217 classification strategies for detecting amyloid-β (Aβ) positivity: standard single cutoff, subgroup-specific optimal cutoff, and double cutoff.

**Design, Setting, and Participants:**

This cohort study was a multicenter cross-sectional study conducted from 2016 to 2023; analyses were completed in 2025. Participants were recruited from multiple memory clinics and community-based cohorts. All participants had amyloid positron emission tomography (PET) imaging, clinical evaluation, and p-tau217 testing with measures of estimated glomerular filtration rate (eGFR), BMI, and hemoglobin. Measurements of p-tau217 were made using UGOT Simoa and Roche Elecsys, and the %p-tau217 ratio was assessed using a tau multianalyte assay (C_2_N Diagnostics LLC).

**Exposures:**

Kidney function (chronic kidney disease [CKD], eGFR <60 mL/min/1.73 m^2^; advanced CKD, eGFR <45 mL/min/1.73 m^2^), underweight (BMI <18.5), obesity (BMI ≥27.5), and anemia (hemoglobin <12 g/dL in women, <13 g/dL in men).

**Main Outcomes and Measures:**

Plasma p-tau217 concentration; standard single cutoff, optimal cutoffs, and double cutoff for Aβ positivity (Centiloid ≥25.5); accuracy; and cost-effectiveness estimated from false-positive, false-negative, and confirmatory imaging costs.

**Results:**

A total of 2571 participants were analyzed with UGOT, 1578 with Roche, and 304 with the C2N %p-tau217 ratio. The mean (SD) age was similar across cohorts (71.3 [8.6] years in the UGOT cohort, 71.3 [8.5] years in the Roche cohort, and 71.8 [7.8] years in the C2N cohort); there were 1633 (63.5%), 1006 (63.8%), and 191 (62.8%) women and 938 (36.5%), 572 (36.2%), and 113 (37.2%) men, respectively. In the UGOT cohort, the optimal cutoff improved diagnostic accuracy compared with the standard single cutoff, particularly in CKD and anemia (CKD: from 0.65; 95% CI, 0.57-0.72; to 0.83; 95% CI, 0.76-0.89; anemia: from 0.80; 95% CI, 0.76-0.84; to 0.86; 95% CI, 0.82-0.90), with consistent findings in the Roche cohort. In all biological subgroups, the double-cutoff strategy also increased accuracy relative to the single cutoff and reduced false classifications but yielded 12% to 39% intermediate results. When compared directly, the optimal cutoff provided higher accuracy than the double cutoff for CKD while lowering total diagnostic costs. For anemia, the double cutoff showed slightly higher accuracy but required confirmatory PET in up to 25% of cases, offsetting its economic advantage. In obesity, the double cutoff remained superior for both diagnostic accuracy and cost efficiency.

**Conclusions and Relevance:**

This study found that both the optimal cutoff and the double-cutoff strategy outperformed the standard single cutoff, each showing distinct strengths across subgroups. These findings support biologically informed thresholds to improve diagnostic accuracy and cost efficiency when implementing plasma p-tau217.

## Key Points

**Question:**

For plasma phosphorylated tau 217–based amyloid-β detection, does the strategy of using biological subgroup–specific optimal single cutoffs or a double cutoff better optimize diagnostic accuracy and cost efficiency?

**Findings:**

This cohort study found that subgroup-specific optimal cutoffs improved accuracy over the standard single cutoff, especially in chronic kidney disease (CKD) and anemia. Compared with a double cutoff, the optimal cutoff had similar or better accuracy in CKD with lower cost, whereas a double cutoff was slightly better in underweight and anemia but created intermediates; in obesity, a double cutoff remained superior.

**Meaning:**

Biologically optimized cutoffs offer a balanced, cost-efficient default, particularly in CKD and anemia, while a double cutoff retains advantages in obesity.

## Teaser

This cohort study compares the performance of 3 plasma p-tau217 classification strategies for detecting amyloid-β positivity to consider which offers better optimize diagnostic accuracy and cost efficiency.

## Introduction

Plasma phosphorylated tau (p-tau) 217 has emerged as a promising blood biomarker for detecting amyloid-β (Aβ) positron emission tomography (PET) positivity in Alzheimer disease (AD),^1,2,3,4,5^ leading to growing anticipation of its clinical application. However, to implement p-tau217 in practice, it is essential to define the optimal cutoffs that maximize diagnostic accuracy for specific clinical cohorts. Plasma p-tau217 levels are influenced by several biological factors and comorbidities, including body mass index (BMI), hemoglobin level, estimated glomerular filtration rate (eGFR), and cardiovascular conditions such as myocardial infarction or stroke.^6,7,8^ Although a recent study quantitatively examined the effect of chronic kidney disease (CKD) on p-tau217 levels,^9^ it remains unclear how these biological factors, and their adjusted cutoffs, affect the diagnostic accuracy for detecting Aβ PET positivity.

In addition to biological variability, clinical implementation also requires balancing diagnostic precision with economic feasibility. Biological heterogeneity may not only influence p-tau217 levels but also affect the cost-effectiveness of different diagnostic strategies by altering the frequency of false classifications and indeterminate results. Recent frameworks, such as the double-cutoff strategy, have been proposed to reduce misclassification risk by defining “gray zones” that minimize false positives and negatives^10,11,12^; however, this approach increases the proportion of indeterminate cases and necessitates confirmatory PET scans, thereby raising total diagnostic costs.^13^ Instead, biological subgroup–specific optimal cutoffs might be the cost-effective strategies that maintain diagnostic accuracy while accounting for biological variability and minimizing unnecessary confirmatory testing for real-world translation of p-tau217 testing.

Therefore, in this study, we aimed to determine how 3 biological factors, kidney dysfunction, BMI, and anemia (based on the Kidney Disease: Improving Global Outcomes [KDIGO], World Health Organization [WHO], and Asian-specific BMI criteria),^14,15,16^ affect the optimal cutoff and diagnostic accuracy of p-tau217 for detecting Aβ PET positivity. We used 3 platforms: the Simoa assay at University of Gothenburg (participants hereafter referred to as the UGOT cohort), the Elecsys assay at Roche Diagnostics (Roche cohort), and p-tau217/nonphosphorylated-tau217 ratio (%p-tau217), a normalized ratio that is part of a tau multiple analyte (6-plex) assay developed by C_2_N Diagnostics (C2N cohort). We further evaluated whether these subgroup-specific and double cutoffs improve diagnostic accuracy and cost-effectiveness compared with the standard single cutoff.

## Methods

### Participants

A total of 2571 individuals from the Korea Registries to Overcome dementia and Accelerate Dementia (K-ROAD) research cohort were enrolled in this study.^17,18^ All participants were ethnically Korean, identified as East Asian, and included individuals with cognitively unimpaired status, mild cognitive impairment,^19^ and dementia of Alzheimer type.^20^ A comprehensive neuropsychological assessment was performed using the Seoul Neuropsychological Screening Battery (eTable 1 in Supplement 1).^21^ Detailed inclusion and exclusion criteria are described in the eMethods in Supplement 1.

This study received approval from the institutional review board of Samsung Medical Center (No. 2021-02-135). All participants provided written informed consent in accordance with the Declaration of Helsinki. Additionally, the study adhered to the Strengthening the Reporting of Observational Studies in Epidemiology (STROBE) reporting guidelines.

### Aβ PET Acquisition and Quantification

For both 18F-florbetaben and 18F-flutemetamol PET, a dynamic emission PET scan lasting 20 minutes (divided into four 5-minute segments) was acquired 90 minutes after the injection of an average dose of 311.5 MBq of 18F-florbetaben or 197.7 MBq of 18F-flutemetamol. Aβ uptake was quantified using the regional direct comparison Centiloid (rdcCL) approach, previously developed in our study to standardize 18F-florbetaben and 18F-flutemetamol PET tracers without requiring^11^ C-labeled Pittsburgh compound B images.^22^ Aβ positivity on PET was determined based on a global magnetic resonance imaging–derived rdcCL cutoff of 25.5.^23,24^ Details are provided in the eMethods in Supplement 1.

### Plasma Collection and Processing

Blood (8 mL) was collected in 0.5-M EDTA tubes, gently mixed for 5 minutes, and centrifuged at 1300 × g for 10 minutes. Plasma was aliquoted into 5-10 vials (0.3 mL each) and stored at –75 °C until analysis, following the National Biobank of Korea guidelines. The interval between plasma collection and Aβ PET ranged from 0 to 69 days (mean, 4 days).

Samples analyzed on the UGOT platform were shipped on dry ice to the UGOT, thawed on wet ice, and centrifuged at 500 × g for 5 minutes at 4 °C. The ALZPath p-tau217 assay (ALZPath) was used, with all analyses performed blinded to clinical data using a single reagent batch. The assay showed excellent analytical performance (intra-assay coefficient of variability, 7.9%; repeatability, 4.0%-8.7%; intermediate precision, 3.5%-10.7%), consistent with Ashton et al.^25^ Samples analyzed with the Roche platform were processed using the automated electrochemiluminescence immunoassay system under the NeuroToolKit initiative.^26^ The prototype Roche Phospho-Tau(217) plasma was measured using the exploratory Roche NeuroToolKit assays, a panel of automated, robust prototype immunoassays (Roche Diagnostics International). Analyses were performed according to the manufacturer’s standard protocol with all personnel blinded to clinical data. A subset of samples was analyzed using a novel mass-spectrometry–based multiple analyte (6-plex) assay, which simultaneously quantifies p-tau217, p-tau181, p-tau205, and their nonphosphorylated counterparts (np-tau), enabling calculation of %p-tau (p-tau ÷ np-tau ratio × 100) to evaluate biologically normalized signals.

### Group Categorization Based on Biological Factors Affecting Plasma p-tau217 Level

Our approach to identifying biological factors affecting p-tau217 was data-driven. We systematically screened more than 20 comorbidities and clinical factors for their associations with plasma p-tau217 after adjusting for Aβ burden (global rdcCL), as detailed in a previous publication.^27^ This unbiased screening identified 5 factors that maintained significant associations with p-tau217 after Aβ adjustment: CKD, underweight status, obesity, hemoglobin levels, and eGFR. Based on these findings, we focused our analysis on 3 biological domains with the strongest and most consistent effects. Kidney function was estimated using the eGFR, calculated by the Chronic Kidney Disease Epidemiology Collaboration equation^28^ based on age, sex, and serum creatinine levels measured enzymatically on automated chemistry analyzers (Roche Cobas or Beckman Coulter). Participants were stratified into non-CKD and CKD by an eGFR less than 60 mL/min/1.73 m^2^ per KDIGO guidelines,^14^ and further categorized into 2 subgroups: eGFR less than 45 mL/min/1.73 m^2^ and eGFR 45 to 60 mL/min/1.73 m^2^. BMI was calculated as weight in kilograms divided by height in meters squared and categorized as underweight (<18.5), normal (≥18.5 and <27.5), and obese (≥27.5), adopting WHO classification with an Asian-specific cutoff for obesity.^15^ For practical purposes, the normal (18.5-22.9) and overweight (23-27.5) categories were combined. Anemia was defined by WHO criteria: hemoglobin less than 12.0 g/dL in women and less than 13.0 g/dL in men.^16^ Coronary artery disease, including myocardial infarction and angina, and stroke were defined as physician-diagnosed conditions documented in medical records or confirmed self-reported histories verified through medical review.

### Cost-Based Evaluation of Misclassification Under Different p-tau217 Cutoff Strategies

To evaluate the clinical implications, we performed a cost-based analysis of misclassification. For each assay and strategy, we calculated the number of false negatives, false positives, and intermediate cases. In the double-cutoff model, intermediate cases were assumed to undergo confirmatory Aβ PET at a cost of $4000 per scan.^29^ For false-positive cases, we assumed unnecessary administration of Aβ-targeted therapy, with a cost of $30 000 per person, based on the 18-month wholesale price of anti-Aβ monoclonal antibodies (eg, lecanemab),^30^ under the assumption that anti-Aβ therapy is applicable across all cognitive stages. For false-negative cases, we estimated an opportunity cost of $8000 per person, assuming they would miss the clinical benefit of Aβ-targeted therapy under the same assumption. This was based on lecanemab’s estimated gain of 0.08 quality-adjusted life-years and a $100 000 willingness-to-pay threshold.^31,32^

### Statistical Analysis

Continuous variables were summarized as mean (SD), categorical as counts (%), and plasma p-tau217 as median (IQR) because of nonnormality. Plasma p-tau217 values exceeding 3 SDs were removed as outliers. The relationships between plasma p-tau217 and biological factors (eGFR, BMI, hemoglobin) were visualized using the generalized additive model curves. For the association between p-tau217 and categorized biological factors, linear regression analyses were performed adjusting for age and sex (model 1) and additionally for global Aβ uptake (rdcCL) on PET (model 2).

We obtained the “standard” single cutoff of p-tau217 for detecting Aβ positivity in the total group. The standard single cutoff was determined through receiver operating characteristic analysis by maximizing the Youden index. The standard double cutoff were determined as previously described: the upper cutoff at approximately 95% specificity (maximizing sensitivity) and the lower at approximately 95% sensitivity (maximizing specificity). Confidence intervals were estimated using 1000 bootstrap resamples (2.5th-97.5th percentiles). Participants were classified as positive, negative, or intermediate according to their p-tau217 values relative to these cutoffs. Within each biological subgroup, “optimal” cutoffs for detecting Aβ positivity were determined through receiver operating characteristic analysis.

Diagnostic performance metrics including sensitivity, specificity, positive predictive value, negative predictive value, and accuracy were calculated for standard single and double cutoffs derived from the overall population and optimal cutoffs. For the double-cutoff approach, intermediate cases were excluded from accuracy calculations and reported separately. The diagnostic accuracies of the standard single cutoff and the optimal cutoff were compared using the McNemar test.

All tests were 2-sided with statistical significance set at *P* < .05. Analyses were performed using R version 4.3.2 (R Foundation) and RStudio version 2023.12.0 + 369 (Posit).

## Results

### Characteristics of Study Participants

The UGOT, Roche, and C2N cohorts included 2571, 1578, and 304 participants, respectively (Table 1). The mean (SD) age was similar across cohorts (71.3 [8.6], 71.3 [8.5], and 71.8 [7.8] years, respectively); there were 1633 (63.5%), 1006 (63.8%), and 191 (62.8%) women and 938 (36.5%), 572 (36.2%), and 113 (37.2%)men, respectively. *APOE* ε4 carrier frequency was 1015 participants (39.6%), 602 participants (38.2%), and 118 participants (39.3%) in the UGOT, Roche, and C2N cohorts, respectively. Diagnostic distributions (cognitively unimpaired, mild cognitive impairment, and dementia of Alzheimer type) were as follows: UGOT cohort: 633 participants (24.6%), 1344 (52.3%), 594 (23.1%); Roche cohort: 421 (26.7%), 850 (53.9%), 307 (19.5%); and C2N cohort: 104 (34.2%), 149 (49.0%), 51 (16.8%), respectively. Aβ PET positivity was observed in 1346 participants (52.4%) in the UGOT cohort, 727 (46.1%) in the Roche cohort, and 184 (60.5%) in the C2N cohort.

**Table 1.** Baseline Characteristics of Study Participants

Characteristic	Cohort, No. (%)		
	UGOT (n = 2571)	Roche (n = 1578)	C2N (n = 304)
Age, mean (SD), y	71.3 (8.6)	71.3 (8.5)	71.8 (7.8)
Sex			
Female	1633 (63.5)	1006 (63.8)	191 (62.8)
Male	938 (36.5)	572 (36.2)	113 (37.2)
Education level, mean (SD), y	10.7 (4.8)	10.7 (4.7)	11.7 (4.4)
*APOE* ε4 carriers	1015 (39.6)	602 (38.2)	118 (39.3)
Diagnosis			
Cognitively unimpaired	633 (24.6)	421 (26.7)	104 (34.2)
Mild cognitive impairment	1344 (52.3)	850 (53.9)	149 (49.0)
Dementia of Alzheimer type	594 (23.1)	307 (19.5)	51 (16.8)
Vascular risk factors			
Hypertension	1228 (47.8)	785 (49.7)	156 (51.3)
Diabetes	609 (23.7)	421 (26.7)	78 (25.7)
Hyperlipidemia	1216 (47.3)	826 (52.3)	173 (56.9)
Stroke	215 (8.4)	146 (9.3)	29 (9.5)
Coronary artery disease	284 (11.0)	186 (11.8)	42 (13.8)
CKD			
No CKD	2330 (92.6)	1437 (92.0)	271 (96.8)
CKD	187 (7.4)	125 (8.0)	9 (3.2)
Stage 3a (eGFR 45-60 mL/min/1.73 m^2^)	147 (5.8)	101 (6.5)	7 (2.5)
Stage 3b-5 (eGFR <45 mL/min/1.73 m^2^)	40 (1.6)	24 (1.5)	2 (0.7)
BMI^a^			
Underweight	98 (3.8)	58 (3.7)	8 (2.6)
Normal weight	2199 (86.1)	1364 (86.8)	272 (89.5)
Obesity	258 (10.1)	150 (9.5)	24 (7.9)
Anemia^b^	429 (17.1)	283 (18.2)	54 (17.9)
Amyloid PET positivity	1346 (52.4)	727 (46.1)	184 (60.5)
p-tau217 (% p-tau217 for C2N cohort), median (IQR), pg/mL	0.5 (0.3-1.0)	0.3 (0.2-0.7)	4.3 (1.5-8.6)
MMSE score, mean (SD)^c^	22.7 (7.7)	23.4 (7.4)	25.3 (4.7)
CDR-SB score, mean (SD)^d^	2.2 (2.7)	2.1 (2.6)	2.0 (2.2)

Abbreviations: *APOE*, apolipoprotein E; CKD, chronic kidney disease; BMI, body mass index; p-tau217, phosphorylated tau 217; MMSE, Mini-Mental State Examination; CDR-SB, Clinical Dementia Rating sum of boxes; PET, positron emission tomography; %p-tau217, ratio of p-tau217 to nonphosphorylated tau 217 × 100 measured using a mass-spectrometry–based multiple analyte assay; WHO, World Health Organization.

^a^ BMI was calculated as weight in kilograms divided by height in meters squared and categorized as underweight (<18.5), normal (≥18.5 and <27.5), and obese (≥27.5), adopting the WHO classification with an Asian-specific cutoff for obesity.

^b^ Anemia was defined by WHO criteria: hemoglobin <12.0 g/dL in women and <13.0 g/dL in men.

^c^ The MMSE score range is 0 to 30, with higher scores indicating better cognitive function.

^d^ The CDR-SB score range is 0 to 18, where 0 indicates normal cognition and 18 signifies severe dementia.

### Association Between Biological Factors and Plasma p-Tau217 Levels

**Figure 1.**
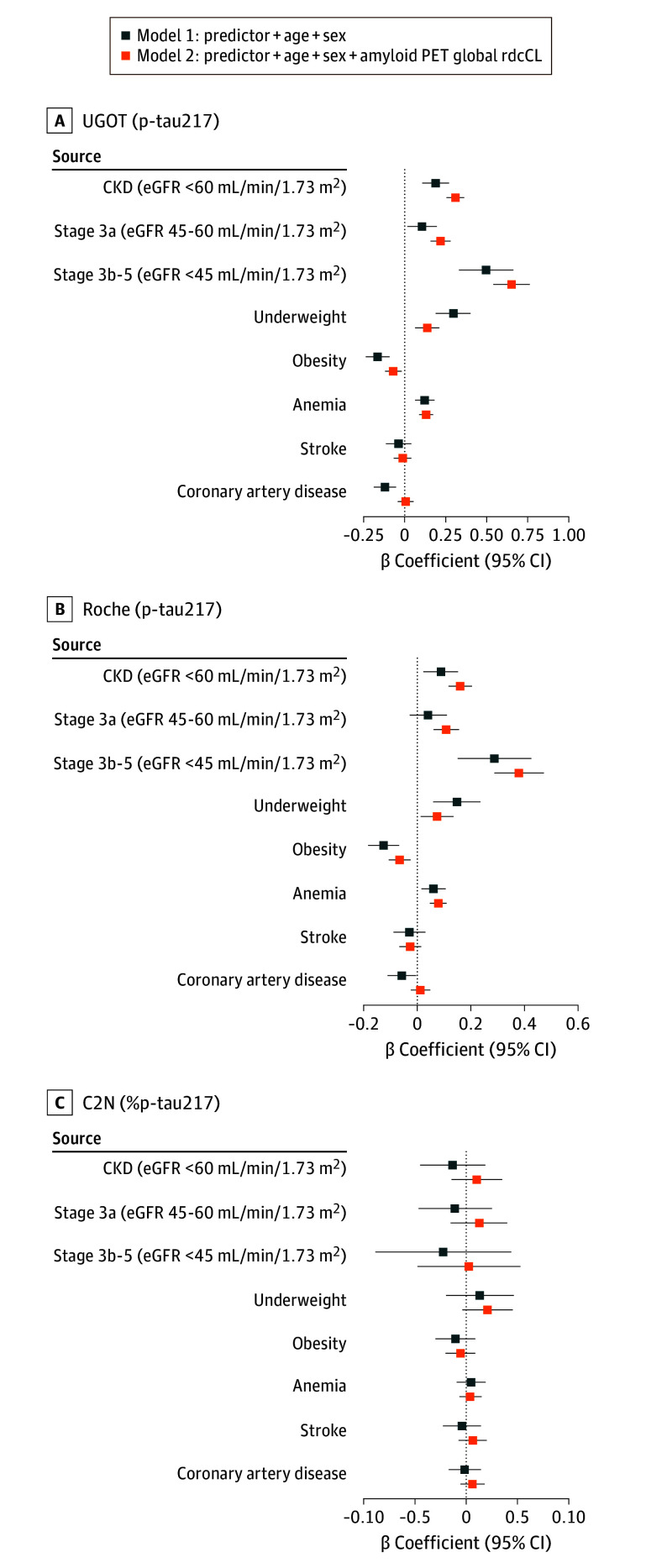
Forest Plots Showing β Coefficients From Linear Regression Analyses Cohorts were named according to the testing platforms: the Simoa assay at University of Gothenburg (UGOT, A), the Elecsys assay at Roche Diagnostics (Roche, B) and a tau multiple analyte (6-plex) assay developed by C_2_N Diagnostics (C2N, C). BMI indicates body mass index; CKD, chronic kidney disease; eGFR, estimated glomerular filtration rate; BMI, body mass index; PET, positron emission tomography; p-tau217, phosphorylated tau 217; %p-tau217, ratio of phosphorylated-tau217 to nonphosphorylated-tau217 × 100 measured using a mass-spectrometry–based multiple analyte assay; rdcCL, regional direct comparison Centiloid approach.

When biological variables were analyzed as continuous factors, plasma p-tau217 levels were inversely associated with eGFR, BMI, and hemoglobin in both Aβ-negative and Aβ-positive individuals in all cohorts (eFigure 1 in Supplement 1). When categorized, participants with CKD, underweight, or anemia exhibited higher plasma p-tau217 levels compared with reference groups, whereas the association with obesity was modest in the UGOT and Roche cohorts. These associations remained significant even after adjusting for Aβ uptakes. In contrast, none of these biological factors were associated with %p-tau217 in the C2N cohort (Figure 1).

Coronary artery disease showed an inverse association with p-tau217 in age- and sex-adjusted models (UGOT: β = −0.120; 95% CI, –0.173 to –0.067; *P* < .001; Roche: β = −0.057; 95% CI, –0.108 to –0.006; *P* = .03), but this became nonsignificant after Aβ adjustment (UGOT: β = 0.006; 95% CI, –0.043 to 0.055; *P* = .81; Roche: β = 0.011; 95% CI, –0.024 to 0.046; *P* = .54). Stroke was not associated with p-tau217 in either unadjusted (UGOT: β = –0.038; 95% CI, –0.112 to 0.036; *P* = .32; Roche: β = –0.030; 95% CI, –0.089 to 0.029; *P* = .32) or Aβ-adjusted models (UGOT: β = –0.013; 95% CI, –0.064 to 0.038; *P* = .62; Roche: β = –0.027; 95% CI, –0.068 to 0.014; *P* = .19). In the C2N cohort, neither coronary artery disease nor stroke was associated with %p-tau217.

### Diagnostic Performance of Standard Single and Double Cutoffs in the Total Cohort

Cutoffs analyses were performed in the UGOT and Roche cohorts. Plasma p-tau217 demonstrated high diagnostic performance for identifying Aβ positivity, with an area under the curve (AUC) of 0.95 (95% CI, 0.94-0.96) in the UGOT cohort and 0.94 (95% CI, 0.93-0.96) in the Roche cohort (eFigure 2, A and B, in Supplement 1). Using the standard single cutoff (UGOT/Roche, 0.44/0.316 pg/mL), overall accuracy reached 0.91 (95% CI, 0.89-0.92)/0.90 (95% CI, 0.89-0.91), with sensitivity and specificity of 0.94 (95% CI, 0.93-0.95)/0.93 (95% CI, 0.91-0.94) and 0.87 (95% CI, 0.84-0.88)/0.88 (95% CI, 0.86-0.90), respectively (Table 2). Applying the standard double cutoff (UGOT/Roche, 0.40-0.75/0.25-0.50), derived from 95% specificity and sensitivity thresholds (eFigure 2, C and D, in Supplement 1), improved diagnostic precision to 0.95 (95% CI, 0.94-0.96)/0.95 (95% CI, 0.94-0.96) accuracy, with sensitivity and specificity of 0.95 (95% CI, 0.94-0.96)/0.96 (95% CI, 0.95-0.98) and 0.95 (95% CI, 0.93-0.96)/0.94 (95% CI, 0.92-0.96), respectively (Table 2). The intermediate cases were 18.7% and 19.9% in the UGOT and Roche cohorts, respectively.

**Table 2.** Diagnostic Performance Metrics of Standard Single Cutoff, Double Cutoff, and Optimal Cutoff Strategies for Detecting Amyloid Positivity

Subgroup	No. of participants	Prevalence, No. (%)^a^	Cutoff type	Threshold, pg/mL	Accuracy (95% CI)	Sensitivity (95% CI)	Specificity (95% CI)	PPV (95% CI)	NPV (95% CI)	Intermediate, %
**UGOT cohort**										
Total	2571	1346 (52.4)	Standard single	0.44	0.91 (0.89-0.92)	0.94 (0.93-0.95)	0.87 (0.84-0.88)	0.88 (0.87-0.90)	0.93 (0.92-0.94)	
			Double	0.40-0.75	0.95 (0.94-0.96)	0.95 (0.94-0.96)	0.95 (0.93-0.96)	0.94 (0.93-0.96)	0.95 (0.94-0.96)	18.7
CKD	126	44 (34.9)	Standard single	0.44	0.65 (0.57-0.72)	0.95 (0.89-1.00)	0.49 (0.39-0.60)	0.50 (0.45-0.56)	0.95 (0.89-1.00)	
			Double	0.40-0.75	0.80 (0.72-0.88)	1.00 (1.00-1.00)	0.65 (0.52-0.77)	0.68 (0.59-0.78)	1.00 (1.00-1.00)	29.4
			Optimal	0.68	0.83 (0.76-0.89)	0.93 (0.86-1.00)	0.77 (0.67-0.85)	0.68 (0.60-0.78)	0.95 (0.91-1.00)	
CKD, eGFR 45-60 mL/min/1.73 m^2^	98	34 (34.7)	Standard single	0.44	0.70 (0.62-0.79)	0.94 (0.85-1.00)	0.58 (0.45-0.70)	0.54 (0.47-0.62)	0.95 (0.88-1.00)	
			Double	(0.40-0.75)	0.85 (0.76-0.94)	1.00 (1.00-1.00)	0.75 (0.60-0.89)	0.74 (0.63-0.87)	1.00 (1.00-1.00)	30.6
			Optimal	0.65	0.88 (0.81-0.94)	0.94 (0.85-1.00)	0.84 (0.75-0.94)	0.76 (0.66-0.89)	0.96 (0.91-1.00)	
CKD, eGFR <45 mL/min/1.73 m^2^	28	10 (35.7)	Standard single	0.44	0.46 (0.39-0.57)	1.00 (1.00-1.00)	0.17 (0.06-0.33)	0.40 (0.37-0.45)	1.00 (0.64-1.00)	
			Double	(0.40-0.75)	0.62 (0.50-0.78)	1.00 (1.00-1.00)	0.27 (0.08-0.55)	0.56 (0.45-0.71)	1.00 (1.00-1.00)	25.0
			Optimal	0.94	0.86 (0.71-0.96)	1.00 (1.00-1.00)	0.78 (0.56-0.94)	0.71 (0.56-0.91)	1.00 (1.00-1.00)	
Underweight^b^	94	68 (72.3)	Standard single	0.44	0.91 (0.86-0.96)	0.97 (0.93-1.00)	0.77 (0.62-0.92)	0.92 (0.86-0.97)	0.91 (0.79-1.00)	
			Double	(0.40-0.75)	0.95 (0.90-0.99)	0.98 (0.95-1.00)	0.85 (0.68-1.00)	0.95 (0.91-1.00)	0.94 (0.83-1.00)	11.7
			Optimal	0.64	0.91 (0.86-0.97)	0.93 (0.85-0.99)	0.88 (0.77-1.00)	0.95 (0.91-1.00)	0.82 (0.70-0.96)	
Obesity^b^	242	98 (40.5)	Standard single	0.44	0.88 (0.84-0.92)	0.91 (0.85-0.96)	0.87 (0.81-0.92)	0.82 (0.77-0.89)	0.93 (0.89-0.97)	
			Double	(0.40-0.75)	0.94 (0.90-0.97)	0.89 (0.81-0.96)	0.97 (0.93-0.99)	0.94 (0.88-0.99)	0.94 (0.90-0.98)	19.8
			Optimal	0.45	0.88 (0.84-0.92)	0.91 (0.85-0.96)	0.87 (0.81-0.92)	0.82 (0.77-0.89)	0.93 (0.89-0.97)	
Anemia^c^	274	123 (44.9)	Standard single	0.44	0.80 (0.76-0.84)	0.98 (0.94-1.00)	0.66 (0.59-0.74)	0.70 (0.66-0.75)	0.97 (0.93-1.00)	
			Double	(0.40-0.75)	0.90 (0.86-0.93)	0.98 (0.95-1.00)	0.82 (0.74-0.89)	0.84 (0.78-0.90)	0.98 (0.95-1.00)	25.9
			Optimal	0.60	0.86 (0.82-0.90)	0.92 (0.87-0.96)	0.81 (0.74-0.87)	0.80 (0.74-0.85)	0.92 (0.88-0.96)	
**Roche cohort**										
Total	1578	727 (46.1)	Standard single	0.316	0.90 (0.89-0.91)	0.93 (0.91-0.94)	0.88 (0.86-0.90)	0.87 (0.85-0.89)	0.93 (0.92-0.95)	
			Double	(0.25-0.50)	0.95 (0.94-0.96)	0.96 (0.95-0.98)	0.94 (0.92-0.96)	0.93 (0.91-0.95)	0.97 (0.96-0.98)	19.9
CKD	96	35 (36.5)	Standard single	0.316	0.70 (0.61-0.78)	0.94 (0.86-1.00)	0.56 (0.43-0.69)	0.55 (0.48-0.63)	0.94 (0.87-1.00)	
			Double	(0.25-0.50)	0.82 (0.73-0.90)	1.00 (1.00-1.00)	0.68 (0.52-0.83)	0.71 (0.60-0.82)	1.00 (1.00-1.00)	31.3
			Optimal	0.512	0.82 (0.75-0.90)	0.83 (0.70-0.94)	0.82 (0.72-0.92)	0.73 (0.63-0.84)	0.89 (0.82-0.96)	
CKD, eGFR 45-60 mL/min/1.73 m^2^	78	28 (35.9)	Standard single	0.316	0.74 (0.65-0.83)	0.93 (0.82-1.00)	0.64 (0.50-0.78)	0.59 (0.51-0.70)	0.94 (0.86-1.00)	
			Double	(0.25-0.50)	0.84 (0.74-0.93)	1.00 (1.00-1.00)	0.73 (0.57-0.89)	0.71 (0.59-0.86)	1.00 (1.00-1.00)	29.5
			Optimal	0.35	0.79 (0.71-0.88)	0.93 (0.82-1.00)	0.72 (0.58-0.86)	0.65 (0.55-0.78)	0.95 (0.87-1.00)	
CKD, eGFR <45 mL/min/1.73 m^2^	18	7 (38.9)	Standard single	0.316	0.50 (0.44-0.67)	1.00 (1.00-1.00)	0.18 (0.09-0.45)	0.44 (0.41-0.54)	1.00 (0.61-1.00)	
			Double	(0.25-0.50)	0.73 (0.60-1.00)	1.00 (1.00-1.00)	0.25 (0.17-1.00)	0.70 (0.54-1.00)	1.00 (1.00-1.00)	38.9
			Optimal	0.662	0.83 (0.67-1.00)	1.00 (1.00-1.00)	0.73 (0.45-1.00)	0.70 (0.54-1.00)	1.00 (1.00-1.00)	
Underweight^b^	54	36 (66.7)	Standard single	0.316	0.91 (0.83-0.98)	0.94 (0.86-1.00)	0.83 (0.61-1.00)	0.92 (0.84-1.00)	0.88 (0.74-1.00)	
			Double	(0.25-0.50)	0.95 (0.88-1.00)	0.97 (0.89-1.00)	0.90 (0.69-1.00)	0.97 (0.90-1.00)	0.90 (0.70-1.00)	25.9
			Optimal	0.358	0.91 (0.81-0.98)	0.92 (0.81-1.00)	0.89 (0.72-1.00)	0.94 (0.87-1.00)	0.84 (0.71-1.00)	
Obesity^b^	137	49 (35.8)	Standard single	0.316	0.88 (0.82-0.93)	0.84 (0.73-0.94)	0.90 (0.83-0.95)	0.82 (0.73-0.91)	0.91 (0.85-0.96)	
			Double	(0.25-0.50)	0.94 (0.89-0.98)	0.87 (0.74-0.97)	0.97 (0.93-1.00)	0.93 (0.84-1.00)	0.95 (0.89-0.99)	25.5
			Optimal	0.294	0.89 (0.83-0.94)	0.90 (0.82-0.98)	0.89 (0.82-0.94)	0.81 (0.73-0.90)	0.94 (0.89-0.99)	
Anemia^c^	214	91 (42.5)	Standard single	0.316	0.83 (0.78-0.88)	0.96 (0.91-0.99)	0.73 (0.66-0.81)	0.73 (0.67-0.79)	0.96 (0.91-0.99)	
			Double	(0.25-0.50)	0.93 (0.89-0.96)	0.99 (0.96-1.00)	0.87 (0.79-0.94)	0.87 (0.82-0.94)	0.99 (0.96-1.00)	25.2
			Optimal	0.447	0.89 (0.85-0.93)	0.89 (0.82-0.96)	0.89 (0.83-0.94)	0.85 (0.79-0.92)	0.92 (0.87-0.96)	

Abbreviations: AUC, area under the curve; BMI, body mass index; CKD, chronic kidney disease; eGFR, estimated glomerular filtration rate; NPV, negative predictive value; PPV, positive predictive value; WHO, World Health Organization.

^a^ Prevalence represents the proportion of participants with amyloid β positron emission tomography positivity within each subgroup.

^a^ BMI was calculated as weight in kilograms divided by height in meters squared and categorized as underweight (<18.5), normal (≥18.5 and <27.5), and obese (≥27.5), adopting the WHO classification with an Asian-specific cutoff for obesity.

^b^ Anemia was defined by WHO criteria: hemoglobin <12.0 g/dL in women and <13.0 g/dL in men.

### Comparative Performance of Optimal and Double Cutoff Strategies Across Biological Subgroups

**Figure 2.**
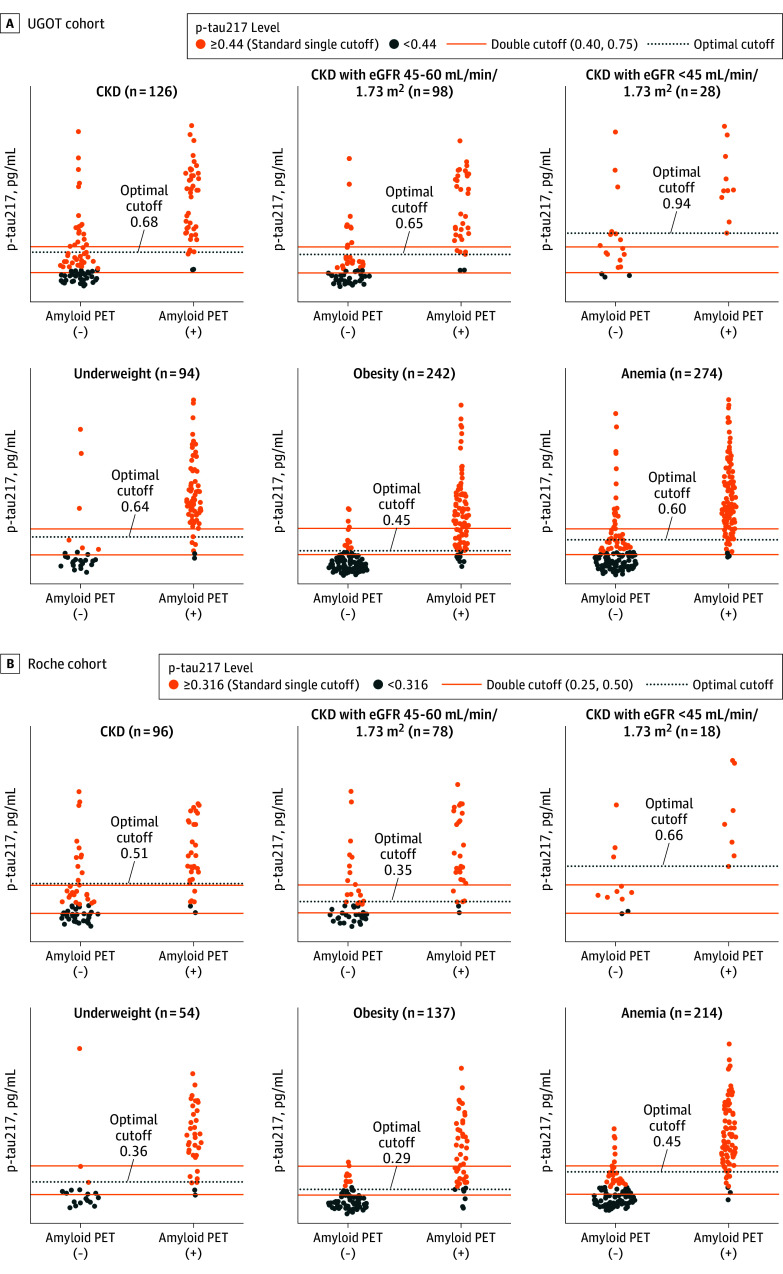
Plasma Phosphorylated Tau 217 (p-Tau217) Cutoff Strategies in Special Populations in the UGOT and Roche Cohorts Points indicate individual patients (dark gray: p-tau217 value below standard single cutoff; orange: p-tau217 value above standard single cutoff). Cohorts were named according to the testing platforms: the Simoa assay at University of Gothenburg (UGOT, A) and the Elecsys assay at Roche Diagnostics (Roche, B). Underweight refers to participants with a body mass index (BMI) less than 18.5 (calculated as weight in kilograms divided by height in meters squared), obesity refers to BMI of 27.5 or greater, and anemia refers to a hemoglobin less than 12 g/dL in women and less than 13 g/dL in men. CKD indicates chronic kidney disease; eGFR, estimated glomerular filtration rate; PET, positron emission tomography.

Cutoff comparisons were only performed in the UGOT/Roche cohorts as %p-tau217 analyzed by C2N platform were not associated with any biological factors. In the UGOT cohort, application of the optimal cutoff markedly reduced false-positive rates in CKD and anemia (−8.0% and −18.3%, respectively), while minimally increasing false-negative rates (0.8% and 2.6%, respectively), compared with the standard single cutoff (Figure 2), resulting in substantial accuracy gains. In CKD, accuracy increased from 0.65 to 0.83 (eGFR <45 mL/min/1.73 m^2^: from 0.46; 95% CI, 0.39-0.57; to 0.86; 95% CI, 0.71-0.96; *P* < .001 by McNemar test), and anemia showed a moderate improvement (from accuracy of 0.80; 95% CI, 0.76-0.84; to 0.86; 95% CI, 0.82-0.90; *P* < .001) (Table 2). Because CKD and anemia frequently co-occurred (odds ratio, 5.12; 95% CI, 3.44-7.63; *P* < .001), we reanalyzed subgroups excluding overlapping abnormalities. Accuracy improvements persisted independently (CKD without anemia: from 0.81; 95% CI, 0.70-0.91; to 0.91; 95% CI, 0.83-0.98; *P* = .004; anemia without CKD: from 0.87; 95% CI, 0.82-0.91; to 0.90; 95% CI, 0.86-0.94; *P* < .001) (eTable 2 in Supplement 1).

**Figure 3.**
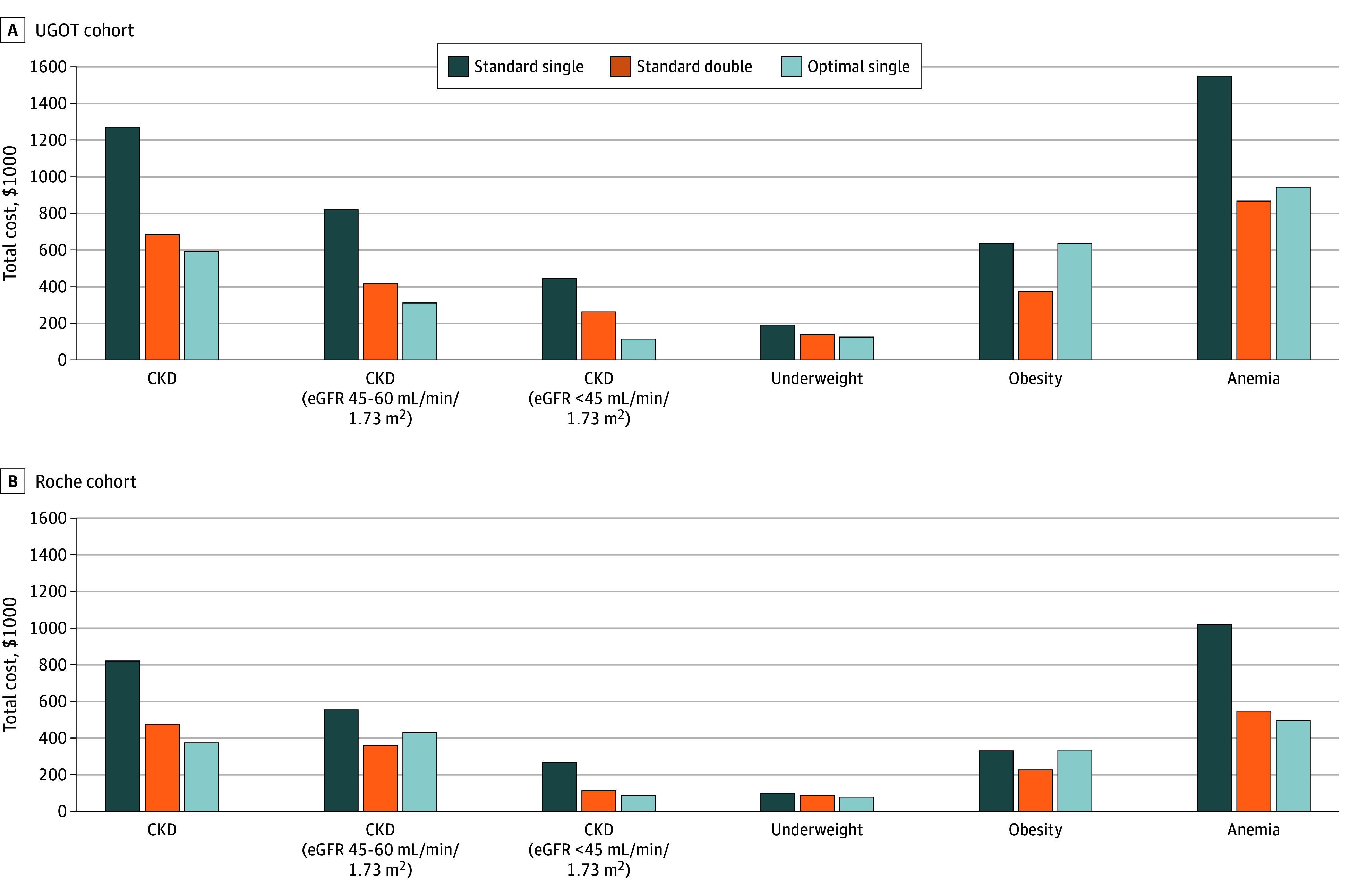
Economic Impact of 3 Phosphorylated Tau 217 Testing Strategies Across Biological Subgroups Total costs per 1000 individuals screened for Alzheimer disease. Cohorts were named according to the testing platforms: the Simoa assay at University of Gothenburg (UGOT, A) and the Elecsys assay at Roche Diagnostics (Roche, B). Cost calculations included false-positive results ($30 000 per patient for unnecessary treatment), false-negative results ($8000 per patient for delayed diagnosis), and intermediate results requiring amyloid positron emission tomography confirmation ($4000 per scan). CKD indicates chronic kidney disease; eGFR, estimated glomerular filtration rate.

The double-cutoff strategy reduced false-positive rates in CKD and anemia (−13.1% and −9.3%) by reallocating many individuals into the intermediate category, while only a small proportion of true positives was shifted to intermediate status (3.2% and 8.4%, respectively) (Figure 2). Although the emergence of an intermediate group (12%-39%) required additional confirmatory testing (Table 2), the overall reduction in false classifications led to lower diagnostic costs (eg, CKD: from $127 600 to $688 000; underweight: from $196 000 to $142 000; obesity: from $642 000 to $376 000; anemia: from $155 400 to $870 000) (Figure 3 and eTable 3 in Supplement 1).

When directly compared with the double cutoff, the optimal cutoff provided higher accuracy in CKD (accuracy of 0.83; 95% CI, 0.76-0.89; vs 0.80; 95% CI, 0.72-0.88; for the double cutoff) and further reduced total costs (from $688 000 to $594 000; advanced CKD: from $268 000 to $120 000) (Figure 3 and eTable 3 in Supplement 1). In anemia and underweight, the double cutoff yielded slightly higher accuracy but required confirmatory PET scans in up to 25% of cases (Table 2), offsetting its diagnostic advantage and increasing total costs. For obesity, the double cutoff remained superior in both accuracy and cost. These trends were consistent in the Roche cohort (Table 2, Figure 2 and Figure 3, and eTable 3 in Supplement 1).

## Discussion

In this study, we evaluated whether optimal cutoffs, adjusted for key biological conditions such as CKD, underweight, obesity, and anemia, could improve Aβ detection beyond standard single cutoffs mainly across 2 analytical platforms. The optimal cutoffs improved diagnostic accuracy compared with the standard single cutoff, particularly in CKD and anemia. Compared with the double-cutoff strategy, the optimal cutoff provided comparable diagnostic precision across subgroups, enhancing accuracy and reducing cost in CKD, maintaining marginal cost benefits in anemia, but showing no gain in obesity, where the double-cutoff strategy remained superior in both diagnostic and economic performance. Taken together, these findings suggest that both the optimal cutoff and the double-cutoff strategy outperformed the standard single cutoff, each showing distinct strengths across subgroups. These findings support biologically informed thresholds to improve diagnostic accuracy and cost efficiency when implementing plasma p-tau217.

In the present study, plasma p-tau217 from UGOT and Roche platforms was inversely associated with eGFR, BMI, and hemoglobin level, and remained higher in participants with CKD, underweight, and anemia (independent of Aβ status) while the association with obesity was modest. In contrast, the %p-tau217 ratio measured using the a mass-spectrometry–based multiple analyte assay showed minimal modulation by kidney function, BMI, or hemoglobin level. These findings support that %p-tau217 may serve as a biologically robust alternative when plasma p-tau217 concentrations are influenced by systemic factors.

The most clinically relevant finding of this study was that the optimal cutoff markedly improved diagnostic accuracy compared with the standard single cutoff, particularly in participants with CKD. Across both the UGOT and Roche platforms, accuracy improved substantially in the CKD group, with absolute gains of approximately 18 percentage points in the UGOT cohort and 12 percentage points in the Roche cohort. This improvement followed a clear severity-dependent pattern, being modest in participants with an eGFR of 45 to 60 mL/min/1.73 m^2^ but much more pronounced in those with eGFR less than 45 mL/min/1.73 m^2^, where accuracy increased by as much as 40 percentage points. A modest but consistent enhancement was also observed in anemia, with gains of about 6 percentage points on both platforms. Because CKD and anemia often co-occurred in our cohort, we examined whether their overlap explained these improvements. Even after excluding individuals with both conditions, the diagnostic advantage of the optimal cutoff remained, indicating that kidney dysfunction and anemia exert independent effects on plasma p-tau217 levels. In contrast, underweight and obesity showed negligible change, suggesting that while body composition may slightly influence plasma p-tau217 concentrations, it exerts minimal impact on diagnostic classification. Collectively, these findings indicate that kidney dysfunction and anemia elevate baseline plasma p-tau217 levels, necessitating modest upward adjustment of diagnostic thresholds to preserve precision for Aβ positivity. This underscores the need to account for systemic biological factors, particularly kidney function and anemia, when interpreting plasma biomarker cutoffs in clinically heterogeneous populations.

Another notable finding was that the double-cutoff strategy provided higher diagnostic accuracy than the standard single cutoff across all subgroups. Despite introducing intermediate classifications, it reduced both false negatives and false positives, resulting in overall lower diagnostic costs. However, compared with the double-cutoff strategy, the optimal cutoff achieved similar or higher accuracy in CKD, particularly in advanced stages, while markedly reducing total costs by eliminating intermediate classifications. In contrast, the double-cutoff approach yielded slightly higher accuracy in anemia and underweight, but the need for confirmatory PET scans in 12% to 26% of intermediate cases offset its analytical advantage and led to higher overall costs. For obesity, the double-cutoff approach outperformed the optimal cutoff in both diagnostic accuracy and cost efficiency, suggesting that the impact of adiposity on plasma p-tau217 levels may differ from that of kidney or hematologic abnormalities. Taken together, these results highlight that both the optimal cutoff and the double-cutoff strategy outperformed the standard single cutoff, each showing distinct strengths across subgroups.

### Strengths and Limitations

One notable strength of this study is its large, well-characterized cohort, which enabled comprehensive evaluation of plasma p-tau217 performance across multiple biological factors. However, several limitations should be noted. First, all possible comorbidities potentially affecting p-tau217 levels could not be examined. Second, our cohort’s ethnic homogeneity precludes examination of ethnic variations in p-tau217 thresholds. Future international studies with external validation, continuous modeling approaches, and diverse populations are essential to determine whether population-specific cutoffs are necessary and to establish the broader applicability of these adjustments.

## Conclusions

This study found that kidney dysfunction and anemia significantly elevated plasma p-tau217 concentrations but not %p-tau217. In these clinical conditions, using plasma p-tau217 necessitates adjusted cutoffs for accurate Aβ detection, while BMI variations, particularly obesity, can be managed with standard p-tau217 cutoffs, particularly using the double cutoff approach. Our findings demonstrate that accounting for biological variability enhances the clinical utility of plasma p-tau217 as a biomarker for AD pathology detection. As an alternative approach, assays that selectively detect brain-derived tau or p-tau217–based ratios, instead of assays with greater peripheral tau binding, may serve as complementary alternatives in contexts where biological variability may influence plasma biomarker interpretation.^33,34^

## Notes

Supplement 1.
eMethods
**eTable 1.** The detailed composition of Seoul Neuropsychological Screening Battery **eTable 2.** Diagnostic performance metrics of standard single-cutoff, double-cutoff, and optimal cutoff in the CKD-only and anemia-only groups **eTable 3.** Economic impact of p-tau217 testing strategies across biological subgroups **eFigure 1.** Scatter plots of association between plasma phosphorylated tau 217 (p-Tau217) levels and biological factors **eFigure 2.** Diagnostic performance and risk stratification of plasma p-tau217 for detecting amyloid positivity
eReferences

Supplement 2. Nonauthor collaborators

Supplement 3. Data sharing statement
