# Supplementary material for: Plasma Phosphorylated Tau 217 Cutoffs for Amyloid Pathology and Kidney Function, Body Mass Index, and Anemia
Source: JAMA Neurol. 2026 Feb 2;83(3):269–79. doi: 10.1001/jamaneurol.2025.5530 (PMC12865699; doi:10.1001/jamaneurol.2025.5530)
Supplement: Supplement 1. — eMethods eTable 1. The detailed composition of Seoul Neuropsychological Screening Battery eTable 2. Diagnostic performance metrics of standard single-cutoff, double-cutoff, and optimal cutoff in the CKD-only and anemia-only groups eTable 3. Economic impact of p-tau217 testing strategies across biological subgroups eFigure 1. Scatter plots of association between plasma phosphorylated tau 217 (p-Tau217) levels and biological factors eFigure 2. Diagnostic performance and risk stratification of plasma p-tau217 for detecting amyloid positivity eReferences [file jamaneurol-e255530-s001.pdf]

Supplemental Online Content

Yun J, Lee J, Shin D, et al; K-ROAD Study Groups. Plasma phosporylated tau 217 cutoffs for amyloid pathology and kidney function, body mass index, and anemia. *JAMA Neurology*. Published online February 2, 2026. doi:10.1001/jamaneurol.2025.5530

eMethods

eTable 1. The detailed composition of Seoul Neuropsychological Screening Battery

eTable 2. Diagnostic performance metrics of standard single-cutoff, double-cutoff, and optimal cutoff in the CKD-only and anemia-only groups

eTable 3. Economic impact of p-tau217 testing strategies across biological subgroups

eFigure 1. Scatter plots of association between plasma phosphorylated tau 217 (p-Tau217) levels and biological factors

eFigure 2. Diagnostic performance and risk stratification of plasma p-tau217 for detecting amyloid positivity

eReferences

This supplemental material has been provided by the authors to give readers additional information about their work.

© 2026 Yun J et al. *JAMA Neurology*.

eMethods

1. Detailed inclusion/exclusion criteria of the K-ROAD cohort

The Korea Registries to Overcome dementia and Accelerate Dementia research cohort (K-ROAD) aims to develop a genotype–phenotype cohort to accelerate the development of novel diagnostic and therapeutic techniques for neurodegenerative diseases, mainly AD and related dementia syndromes, through nationwide collaboration with 21 university-affiliated hospitals in South Korea between 2016 and 2023. All participants in the K-ROAD cohort were Korean, identified as East Asian. A comprehensive neuropsychological assessment was performed using the Seoul Neuropsychological Screening Battery (SNSB). Cognitively unimpaired participants (n=633) were selected based on the following criteria: (1) absence of medical history that is likely to affect cognitive function based on Christensen's health screening criteria[1] and (2) absence of objective cognitive impairment observed after a comprehensive neuropsychological test on any cognitive domain (above the -1.0 standard deviation (SD) of age- and education-matched norms in memory and below -1.5 SD in other cognitive domains).[2] Participants with mild cognitive impairment (n=1344) met the specified criteria[3]: (1) subjective cognitive complaints by the participants or caregiver; (2) objective cognitive impairment in any cognitive domain (below the -1.0 SD of age- and education-matched norms in memory and below -1.5 SD in other cognitive domains); (3) no significant impairment in activities of daily living as assessed by the Korean-Instrumental Activities of Daily Living (K-IADL) scale[4]; and (4) no dementia. Participants diagnosed with dementia of Alzheimer's type (n = 594) fulfilled the National Institute on Aging-Alzheimer's Association diagnostic criteria.[5] All participants were assessed through clinical interviews, neurological examinations, neuropsychological testing, and brain magnetic resonance imaging (MRI). After these evaluations, clinical diagnoses were established by consensus among a multidisciplinary team. All participants underwent Aβ PET with either 18F-florbetaben or 18F-flutemetamol.

All participants underwent blood tests including complete blood count, blood chemistry tests, vitamin B12/folate measurement, syphilis serology, and thyroid function test to rule out the possibility of medical conditions causing cognitive decline, and *APOE* genotyping was performed. Patients were excluded if they had territorial infarctions, brain tumor, or vascular malformation on MRI. Patients with white matter hyperintensities due to radiation injury, multiple sclerosis, vasculitis, or leukodystrophy were also excluded.

© 2026 Yun J et al. *JAMA Neurology*.

2. Aβ PET acquisition and quantification

For both 18F-florbetaben and 18F-flutemetamol PET, a dynamic emission PET scan lasting 20 minutes (divided into four 5-min segments) was acquired 90 minutes post-injection of an average dose of 311.5 MBq of 18F-florbetaben or 197.7 MBq of 18F-flutemetamol. The resulting three-dimensional PET images were reconstructed using the ordered-subset expectation maximization algorithm, generating a 128×128×48 matrix with voxel dimensions of 2×2×3.27 mm. The reconstruction parameters were identical for both tracers, with 4 iterations and 20 subsets.

Aβ uptake was quantified using the regional direct comparison Centiloid (rdcCL) approach, previously developed in our study to standardize 18F-florbetaben and 18F-flutemetamol PET tracers without requiring ^{11}C-labeled Pittsburgh compound B images.[6] Aβ positivity on PET was determined based on a global MRI-derived rdcCL cutoff of 25.5, established through a Gaussian mixture model analysis of 3,753 individuals aged 55 years or older who underwent either 18F-florbetaben or 18F-flutemetamol PET.[7,8] The global MRI-based rdcCL scales for both tracers demonstrated strong discriminative ability, with an area under the curve exceeding 0.9 for visually assessed Aβ PET positivity. All imaging analyses were conducted at the laboratory of Samsung Medical Center, which served as the core center.

© 2026 Yun J et al. *JAMA Neurology*.

eTable 1. The detailed composition of Seoul Neuropsychological Screening Battery

Seoul neuropsychological Screening Battery (SNSB)	Original neuropsychological tests in use	Modification
**Attention**	**Attention**	
Digit span forward	Digit span forward	
Digit span backward	Digit span backward task (Banken, 1985)	
**Language**	**Language**	
BNT	BNT (Kaplan, Goodglass, & Weintraub, 1983)	Modified as a Korean version
**Visuospatial function**	**Visuospatial function**	
RCFT copy	RCFT copy (Rey & Osterrieth, 1993, Beatty et al., 1996)	Same figure
**Memory**	**Memory**	
SVLT immediate recall	AVLT immediate recall	15 items with 5 learning trials in AVLT, 12 items with 3 learning trials in SVLT
SVLT 20 min delayed recall	AVLT 30 min delayed recall	
SVLT recognition	AVLT recognition (Rey, 1964)	
RCFT immediate recall	RCFT immediate recall	Same figure
RCFT 20 min delayed recall	RCFT 30 min delayed recall (Beatty et al., 1996)	
RCFT recognition	RCFT recognition (Meyers and Meyers 1995)	
**Frontal/executive function**	**Frontal/executive function**	
COWAT animal	Category Fluency – animal (Butters, Granholm, Salmon, Grant, & Wolfe, 1987)	
COWAT supermarket	Category Fluency – Vegetables (Butters, Granholm, Salmon, Grant, & Wolfe, 1987)	
COWAT phonemic		
Stroop color reading	Stroop Color and Word Test (SCWT) (Stroop, 1935)	We used correct answers in 120s in color-word condition.
MMSE	MMSE (Folstein, Folstein, & McHugh, 1975)	Modified as a Korean version
CDR	CDR (Berg, 1988)	Same scale

The Boston Naming Test (BNT), the Rey-Osterrieth Complex Figure Test (RCFT), the Seoul Verbal Learning Test (SVLT), Rey The Auditory-Verbal Learning Test (AVLT), a phonemic and semantic Controlled Oral Word Association Test (COWAT), a Stroop test color reading, Mini-mental state examination (MMSE), and clinical dementia rating (CDR)

© 2026 Yun J et al. *JAMA Neurology*.

eTable 2. Diagnostic performance metrics of standard single-cutoff, double-cutoff, and optimal cutoff in the CKD-only and anemia-only groups

Group	N	Aβ Prevalence (n)	Aβ Prevalence (%)	cutoff	threshold	Accuracy	Sensitivity	Specificity	PPV	NPV	Intermediate (%)
CKD only	47	20	42.6%	Standard single	0.43	0.81 (0.70-0.91)	1.00 (1.00-1.00)	0.67 (0.48-0.85)	0.69 (0.59-0.83)	1.00 (1.00-1.00)	-
	47	20	42.6%	Double	(0.40, 0.65)	0.94 (0.85-1.00)	1.00 (1.00-1.00)	0.88 (0.71-1.00)	0.90 (0.77-1.00)	1.00 (1.00-1.00)	25.53
	47	20	42.6%	Optimal	0.65	0.91 (0.83-0.98)	0.90 (0.75-1.00)	0.93 (0.81-1.00)	0.90 (0.77-1.00)	0.93 (0.83-1.00)	-
Anemia only	163	84	51.5%	Standard single	0.44	0.87 (0.82-0.91)	0.96 (0.92-1.00)	0.77 (0.68-0.86)	0.82 (0.76-0.88)	0.95 (0.90-1.00)	-
	163	84	51.5%	Double	(0.40, 0.65)	0.94 (0.89-0.98)	0.97 (0.93-1.00)	0.90 (0.82-0.97)	0.92 (0.86-0.97)	0.96 (0.91-1.00)	20.25
	163	84	51.5%	Optimal	0.59	0.90 (0.86-0.94)	0.90 (0.83-0.96)	0.90 (0.83-0.96)	0.90 (0.85-0.96)	0.90 (0.84-0.96)	-

Abbreviations: CKD, Chronic Kidney Disease; Aβ, β-amyloid; PPV, positive predictive value; NPV, negative predictive value.

*Prevalence represents the proportion of participants with Aβ PET positivity within each subgroup.

© 2026 Yun J et al. *JAMA Neurology.*

eTable 3. Economic impact of p-tau217 testing strategies across biological subgroups

Cohort	Subgroup (N, Prevalence*)	Strategy	FN (N, %)	FP (N, %)	Intermediate (N, %)	Total cost* (1,000 $)
UGOT	CKD (126, 34.9%)	Standard single-cutoff	2 (1.6)	42 (33.3)	0 (0.0)	1276
		Double-cutoff	0 (0.0)	18 (14.3)	37 (29.4)	688
		Optimal cutoff	3 (2.4)	19 (15.1)	0 (0.0)	594
	CKD 45-60 (98, 34.7%)	Standard single-cutoff	2 (2.0)	27 (27.6)	0 (0.0)	826
		Double-cutoff	0 (0.0)	10 (10.2)	30 (30.6)	420
		Optimal cutoff	2 (2.0)	10 (10.2)	0 (0.0)	316
	CKD<45 (28, 35.7%)	Standard single-cutoff	0 (0.0)	15 (53.6)	0 (0.0)	450
		Double-cutoff	0 (0.0)	8 (28.6)	7 (25.0)	268
		Optimal cutoff	0 (0.0)	4 (14.3)	0 (0.0)	120
	Underweight (94, 72.3%)	Standard single-cutoff	2 (2.1)	6 (6.4)	0 (0.0)	196
		Double-cutoff	1 (1.1)	3 (3.2)	11 (11.7)	142
		Optimal cutoff	5 (5.3)	3 (3.2)	0 (0.0)	130
	Obesity (242, 40.5%)	Standard single-cutoff	9 (3.7)	19 (7.9)	0 (0.0)	642
		Double-cutoff	8 (3.3)	4 (1.7)	48 (19.8)	376
		Optimal cutoff	9 (3.7)	19 (7.9)	0 (0.0)	642
	Anemia (274, 44.9%)	Standard single-cutoff	3 (1.1)	51 (18.6)	0 (0.0)	1554
		Double-cutoff	2 (0.7)	19 (6.9)	71 (25.9)	870
		Optimal cutoff	10 (3.6)	29 (10.6)	0 (0.0)	950
Roche	CKD (96, 36.5%)	Standard single-cutoff	2 (2.1)	27 (28.1)	0 (0.0)	826
		Double-cutoff	0 (0.0)	12 (12.5)	30 (31.2)	480

© 2026 Yun J et al. *JAMA Neurology*.

	Optimal cutoff	6 (6.2)	11 (11.5)	0 (0.0)	378
CKD 45-60 (78, 35.9%)	Standard single-cutoff	2 (2.6)	18 (23.1)	0 (0.0)	556
	Double-cutoff	0 (0.0)	9 (11.5)	23 (29.5)	362
	Optimal cutoff	2 (2.6)	14 (17.9)	0 (0.0)	436
CKD<45 (18, 38.9%)	Standard single-cutoff	0 (0.0)	9 (50.0)	0 (0.0)	270
	Double-cutoff	0 (0.0)	3 (16.7)	7 (38.9)	118
	Optimal cutoff	0 (0.0)	3 (16.7)	0 (0.0)	90
Underweight (54, 66.7%)	Standard single-cutoff	2 (3.7)	3 (5.6)	0 (0.0)	106
	Double-cutoff	1 (1.9)	1 (1.9)	14 (25.9)	94
	Optimal cutoff	3 (5.6)	2 (3.7)	0 (0.0)	84
Obesity (137, 35.8%)	Standard single-cutoff	8 (5.8)	9 (6.6)	0 (0.0)	334
	Double-cutoff	4 (2.9)	2 (1.5)	35 (25.5)	232
	Optimal cutoff	5 (3.6)	10 (7.3)	0 (0.0)	340
Anemia (214, 42.5%)	Standard single-cutoff	4 (1.9)	33 (15.4)	0 (0.0)	1022
	Double-cutoff	1 (0.5)	11 (5.1)	54 (25.2)	554
	Optimal cutoff	10 (4.7)	14 (6.5)	0 (0.0)	500

Abbreviations: CKD, Chronic Kidney Disease; Aβ, β-amyloid; FN, false negatives; FP, false positives.

* Cost per 1 patient ($) FN : 8,000, FP : 30,000, Intermediate : 4,000

© 2026 Yun J et al. *JAMA Neurology*.

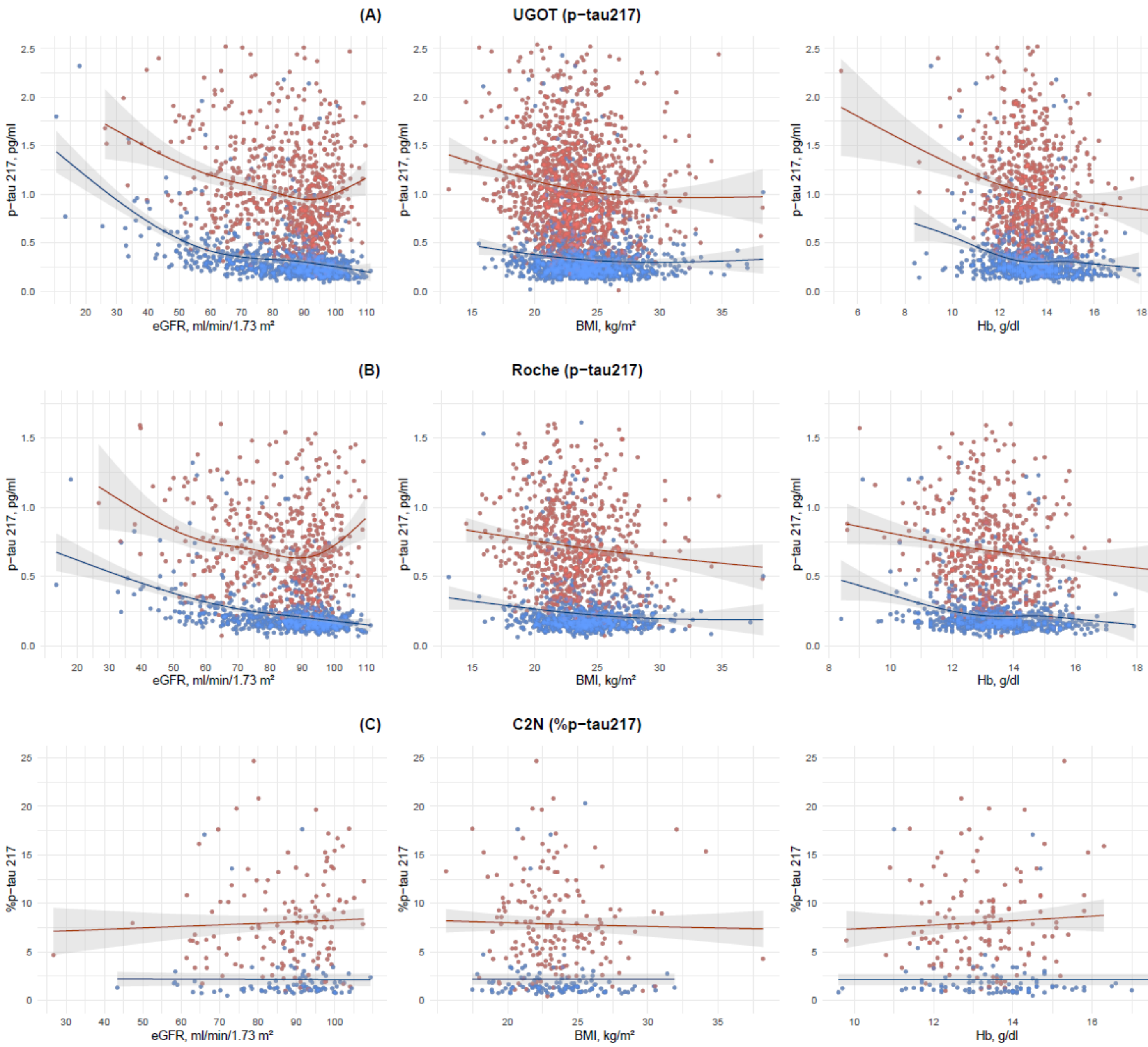

eFigure 1. Scatter plots of association between plasma phosphorylated tau 217 (p-Tau217) levels and biological factors. Scatter plots of plasma p-tau217 versus eGFR, BMI, and hemoglobin for UGOT (A), Roche (B) and C2N (C) platforms. Blue: amyloid negative; red: amyloid-positive participants. Lines show fitted regression with 95% confidence intervals. Abbreviations: eGFR, estimated glomerular filtration rate; BMI, body mass index; Hb, hemoglobin; CKD, chronic kidney disease; p-tau, phosphorylated-tau; %p-tau217, ratio of phosphorylated-tau217 to nonphosphorylated-tau217 x 100 measured using a mass-spectrometry–based multiple analyte assay (C2N).

© 2026 Yun J et al. *JAMA Neurology.*

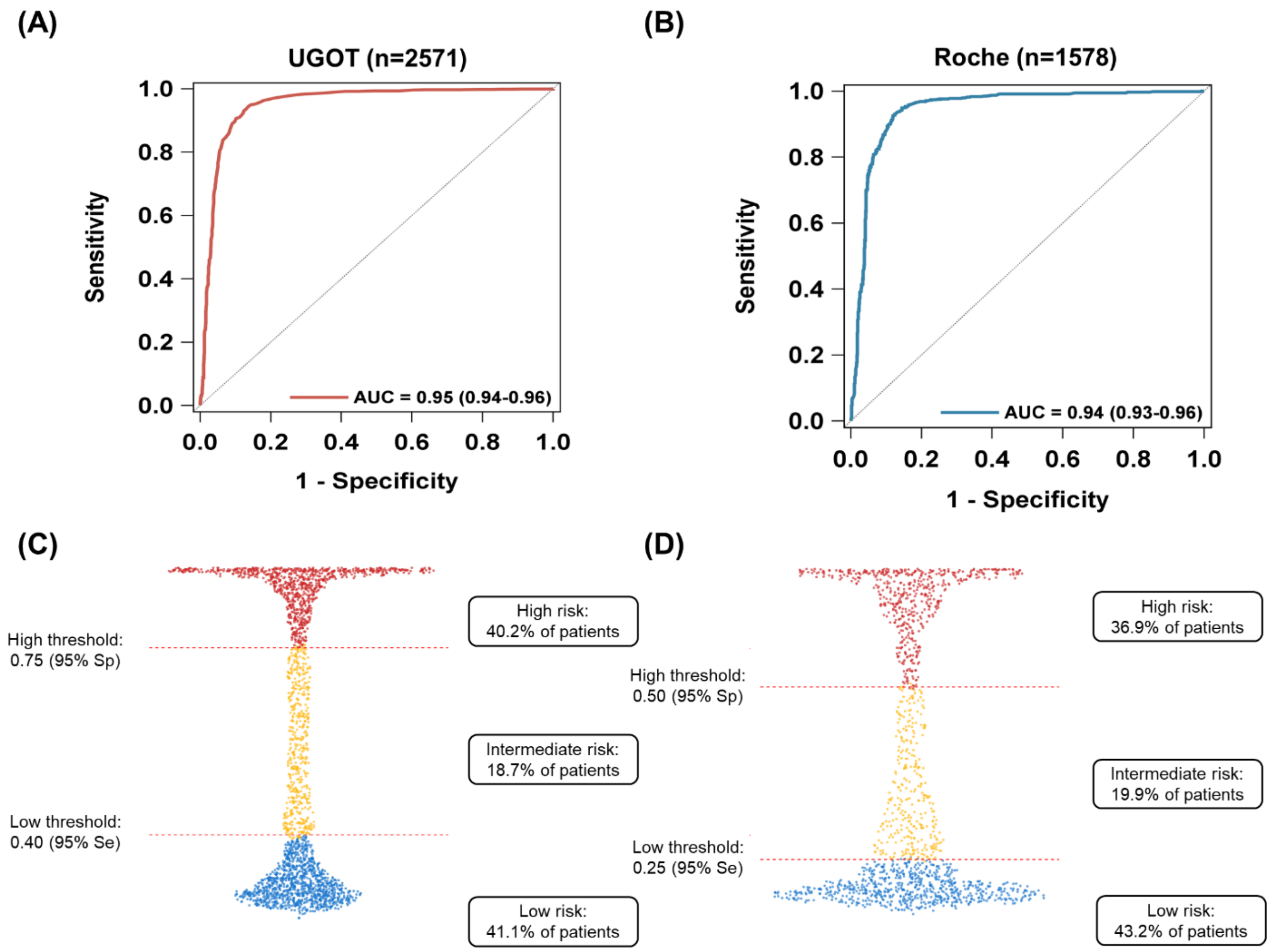

eFigure 2. Diagnostic performance and risk stratification of plasma p-tau217 for detecting amyloid positivity. (A-B) Receiver operating characteristic (ROC) curves for plasma p-tau217 in detecting Aβ-PET positivity using UGOT (A, n=2571) and Roche (B, n=1578) platforms. Area under the curve (AUC) values with 95% confidence intervals are shown. (C-D) Risk stratification using double-cutoff strategy for UGOT (C) and Roche (D). Participants are classified into three categories based on plasma p-tau217 levels: high risk (red, above high threshold), intermediate risk (yellow, between thresholds), and low risk (blue, below low threshold). Thresholds were optimized for 95% sensitivity (Se) and 95% specificity (Sp). The percentage of patients in each risk category is indicated.

Abbreviations: AUC, area under the curve; CI, confidence interval; Se, sensitivity; Sp, specificity.

© 2026 Yun J et al. *JAMA Neurology.*

eReferences

1. Christensen KJ, Multhaup KS, Nordstrom S, Voss K. A cognitive battery for dementia: Development and measurement characteristics. *Psychol Assess*. 1991;3(2):168-174.

2. Ahn HJ, Chin J, Park A, et al. Seoul Neuropsychological Screening Battery-dementia version (SNSB-D): a useful tool for assessing and monitoring cognitive impairments in dementia patients. *J Korean Med Sci*. 2010;25(7):1071-1076. doi:10.3346/jkms.2010.25.7.1071

3. Albert MS, DeKosky ST, Dickson D, et al. The diagnosis of mild cognitive impairment due to Alzheimer's disease: Recommendations from the national institute on aging-Alzheimer's association workgroups on diagnostic guidelines for Alzheimer's disease. *Focus (Madison)*. 2013;11(1):96-106. doi:10.1176/appi.focus.11.1.96

4. Chin J, Park J, Yang SJ, et al. Re-standardization of the Korean-Instrumental Activities of Daily Living (K-IADL): Clinical Usefulness for Various Neurodegenerative Diseases. *Dement Neurocogn Disord*. 2018;17(1):11-22. doi:10.12779/dnd.2018.17.1.11

5. McKhann GM, Knopman DS, Chertkow H, et al. The diagnosis of dementia due to Alzheimer's disease: recommendations from the National Institute on Aging-Alzheimer's Association workgroups on diagnostic guidelines for Alzheimer's disease. *Alzheimers Dement*. 2011;7(3):263-269. doi:10.1016/j.jalz.2011.03.005

6. Cho SH, Choe YS, Kim HJ, et al. A new Centiloid method for (18)F-florbetaben and (18)F-flutemetamol PET without conversion to PiB. *Eur J Nucl Med Mol Imaging*. 2020;47(8):1938-1948. doi:10.1007/s00259-019-04596-x

7. Kim SJ, Ham H, Park YH, et al. Development and clinical validation of CT-based regional modified Centiloid method for amyloid PET. *Alzheimers Res Ther*. 2022;14(1):157. doi:10.1186/s13195-022-01099-0

8. Park S, Kim K, Yoon S, et al. Establishing Regional Abeta Cutoffs and Exploring Subgroup Prevalence Across Cognitive Stages Using BeauBrain Amylo((R)). *Dement Neurocogn Disord*. 2025;24(2):135-146. doi:10.12779/dnd.2025.24.2.135

© 2026 Yun J et al. *JAMA Neurology.*
