# Supplementary material for: Plasma Phosphorylated Tau 217 Cutoffs for Amyloid Pathology and Kidney Function, Body Mass Index, and Anemia
Source: JAMA Neurol. 2026 Feb 2;83(3):269–79. doi: 10.1001/jamaneurol.2025.5530 (PMC12865699; doi:10.1001/jamaneurol.2025.5530)
Supplement: Supplement 2. — Nonauthor collaborators [file jamaneurol-e255530-s002.pdf]

Supplemental Online Content: Nonauthor Collaborators
*First name, last name, and suffix (if applicable) are required and will appear in PubMed.

*Group Name(s): K-ROAD							
***First Name and Middle Initial(s)**	***Last Name**	***Suffix (eg, Jr, III)**	Academic Degrees	Institution	Location (city, state/province, country)	Role or Contribution, eg, chair, principal investigator	Group (if more than 1 Group listed in the byline) and/or Subgroup (eg, Steering Committee)
Youngsoo	Kim		PhD				K-ROAD
Sun-Ho	Han		PhD				K-ROAD
JoonKyung	Seong		PhD				K-ROAD
Jun-Kyu	Choi		PhD				K-ROAD
Eek-Sung	Lee		MD, PhD				K-ROAD
Tak-Kyeong	Lee		MD, PhD				K-ROAD
Juhee	Chin		PhD				K-ROAD
Chi-Hun	Kim		MD, PhD				K-ROAD
Hee Jin	Kim		MD, PhD				K-ROAD
Haesook	Bok		MS				K-ROAD
Hang-Rai	Kim		MD, PhD				K-ROAD
Seung Joo	Kim		MD				K-ROAD
Seunghee	Na		MD				K-ROAD
Geon Ha	Kim		MD, PhD				K-ROAD
Jin San	Lee		MD, PhD				K-ROAD
Hanna	Cho		MD, PhD				K-ROAD
Byeong C.	Kim		MD, PhD				K-ROAD
Dong Young	Lee		MD, PhD				K-ROAD
So Young	Moon		MD, PhD				K-ROAD
Min Soo	Byun		MD, PhD				K-ROAD
Giijung	Jung		RN, PhD				K-ROAD
Dahyun	Yi		PhD				K-ROAD
Han Na	Lee		RN				K-ROAD
Jae-Won	Jang		MD, PhD				K-ROAD
Jee Hyang	Jeong		MD, PhD				K-ROAD
Young Hee	Jung		MD, PhD				K-ROAD
Jong Hun	Kim		MD, PhD				K-ROAD
Youngju	Kim		MA				K-ROAD

Supplemental Online Content: Nonauthor Collaborators
*First name, last name, and suffix (if applicable) are required and will appear in PubMed.

***First Name and Middle Initial(s)**	***Last Name**	***Suffix (eg, Jr, III)**	Academic Degrees	Institution	Location (city, state/province, country)	Role or Contribution, eg, chair, principal investigator	Group (if more than 1 Group listed in the byline) and/or Subgroup (eg, Steering Committee)
Bo Kyoung	Cheon		PhD				K-ROAD
Jinkyu	Seo		None				K-ROAD
Young	Noh		MD, PhD				K-ROAD
Hyunjung	Yang		CCRC				K-ROAD
Youngji	Ha		RN, CCRC				K-ROAD
Hae-Eun	Shin		None				K-ROAD
Kyunghun	Kang		MD, PhD				K-ROAD
SungHui	Eom		Bachelor's degree in psychology				K-ROAD
Sun-Ho	Han		PhD				K-ROAD
Ki Young	Shin		PhD				K-ROAD
Yeongshin	Kim		PhD				K-ROAD
Jisung	Jang		PhD				K-ROAD
Changsik	Yoon		MS				K-ROAD
Do kyung	Lee		MS				K-ROAD
JoonKyung	Seong		PhD				K-ROAD
Hongki	Ham		MS				K-ROAD
Yu Hyun	Park		PhD				K-ROAD
Soo-Jong	Kim		MS				K-ROAD
Byunghyun	Byun		MD, PhD				K-ROAD
Yejoo	Choi		MS				K-ROAD
Na Kyung	Lee		PhD				K-ROAD
Hong-Hee	Won		PhD				K-ROAD
Minyoung	Cho		MS				K-ROAD
Sang-Hyuk	Jung		PhD				K-ROAD
Dong Hyun	Lee		MD				K-ROAD
Beomsu	Kim		BS				K-ROAD
