# Supplementary material for: Plasma Phosphorylated Tau 217 Cutoffs for Amyloid Pathology and Kidney Function, Body Mass Index, and Anemia
Source: JAMA Neurol. 2026 Feb 2;83(3):269–79. doi: 10.1001/jamaneurol.2025.5530 (PMC12865699; doi:10.1001/jamaneurol.2025.5530)
Supplement: Supplement 3. — Data sharing statement [file jamaneurol-e255530-s003.pdf]

Data Sharing Statement

Yun. Plasma Phosphorylated Tau 217 Cutoffs for Amyloid Pathology and Kidney Function, Body Mass Index, and Anemia. *JAMA Neurol*. Published February 02, 2026.
doi:10.1001/jamaneurol.2025.5530

Data
Data available: No

Additional Information
Explanation for why data not available: The anonymized data for the analyses presented in this report are available upon request from the corresponding authors.
